# Myeloid-Specific Deletion of *Lnx2* Attenuates Estrogen-Deficiency-Induced Bone Loss by Inhibiting Osteoclastogenesis via the NUMB/NOTCH2 Axis

**DOI:** 10.3390/biomedicines14061180

**Published:** 2026-05-22

**Authors:** Wei Wang, Jinhui Zhao, Ang Li, Chen Chen, Weitao Jia, Xiaolin Li

**Affiliations:** 1 Department of Orthopedic Surgery, Shanghai Sixth People’s Hospital Affiliated to Shanghai Jiao Tong University School of Medicine, Shanghai 200233, China; xiaowangwei_ok@126.com (W.W.); bonezhaojinhui@163.com (J.Z.); liang_1@sjtu.edu.cn (A.L.); sjtuchenchen@sjtu.edu.cn (C.C.); 2 Department of Orthopedic Surgery, Shanghai Institute of Microsurgery on Extremities, Shanghai Sixth People’s Hospital Affiliated to Shanghai Jiao Tong University School of Medicine, Shanghai 200233, China

**Keywords:** *Lnx2*, NUMB, NOTCH2, osteoclast, bone resorption, osteoporosis

## Abstract

**Background:** We previously reported that knocking down the ubiquitin E3 ligase LNX2 in bone marrow monocytes by shRNAs attenuated osteoclastogenesis in vitro. However, the role of LNX2 in the regulation of osteoclasts and bone homeostasis in vivo remains unknown. **Methods:** In this study, we generated myeloid *Lnx2* conditional knockout mice by crossing *Lnx2*-flox mice with *LysM*-Cre mice. The role of LNX2 was verified through in vitro osteoclast induction experiments using mononuclear macrophages and experiments on estrogen-deficient osteoporosis models. **Results:** Micro-CT and histological analysis unveiled that loss of *Lnx2* in osteoclast precursor cells decreased osteoclast numbers and increased trabecular bone mass in mice. Moreover, *Lnx2* deficiency prevented bone loss in an ovariectomized mouse model of postmenopausal osteoporosis. In vitro mechanistic studies identified that the loss of *Lnx2* had little effect on cell proliferation but significantly inhibited the formation of osteoclasts and bone resorption. Furthermore, the deletion of *Lnx2* decreased the expression of NOTCH2 and its downstream HES1 via enhancing the level of the NOTCH2 inhibitor, NUMB. **Conclusions:** Our findings elucidate an important role of *Lnx2* in the regulation of osteoclasts and bone metabolism and indicate that *Lnx2* is a potential therapeutic target for the treatment of osteoporosis.

## 1. Introduction

Bone metabolism and homeostasis depend on a dynamic balance between bone resorption by osteoclasts and bone formation by osteoblasts. An imbalance in these activities contributes to the development of various skeletal disorders [1,2]. Osteoporosis is a common clinical disease, especially in elderly female patients [3], and manifests as back and whole-body pain, spinal deformity, and frequent fractures, which seriously affect patient quality of life and impose heavy burdens on individuals, families, and society [4]. Excessive osteoclast-mediated bone loss is the major culprit of osteoporosis.

Osteoclasts are derived from mononuclear precursors of the monocyte/macrophage lineage of hematopoietic stem cells. Under the control of two indispensable cytokines, the macrophage colony-stimulating factor (M-CSF) and receptor activator of NF-κB ligand (RANKL), bone marrow monocytes (BMMs) first differentiate into mononuclear osteoclast precursors, which eventually fuse to form multinucleated mature osteoclasts [2]. M-CSF stimulates the proliferation of monocytes and maintains the survival of osteoclasts by activating the ERK and PI3K/AKT pathways. Osteoclast differentiation is the process by which osteoclast precursors differentiate into mature osteoclasts. The core regulatory pathway is the RANKL/RANK/OPG signaling axis, involving key pathways such as NF-κB, MAPK, and Ca^2+^-NFATc1. These signals, by regulating gene expression and cellular functions, ultimately promote the formation of osteoclasts and bone resorption activity [5,6,7]. However, the mechanism of bone resorption is complex, and an increasing number of other signaling pathways have received widespread attention.

The NOTCH signaling pathway plays a distinctive role in both skeletal development and the preservation of bone homeostasis [8,9]. In vivo studies using mouse models with enhanced and reduced NOTCH function have shown that NOTCH enhances the proliferation of osteoblast precursors while suppressing their final differentiation, resulting in reduced bone formation and osteoporosis [10]. Nevertheless, studies performed by other investigators have reported that NOTCH promotes osteoblast differentiation and the generation of woven bone [11]. Blocking NOTCH signaling in the myeloid lineage decreases bone resorption through inhibition of osteoclast activity, whereas activating NOTCH signaling, especially NOTCH2, enhances osteoclast differentiation [12]. These discrepancies underscore the need for further investigation into the context-dependent regulatory functions of NOTCH signaling in bone cells.

The NOTCH signaling pathway regulates cell proliferation and differentiation. The regulation of NOTCH expression (NOTCH1–4) and modifications endows this pathway with a variety of functions [12]. In the skeleton, the NOTCH signaling directly or indirectly regulates both osteoblast and osteoclast differentiation [13]. NOTCH1 overexpression enhances fracture healing without altering osteoclast numbers and induces hyperosteogeny in zebrafish [14,15], whereas NOTCH2 can directly induce osteoclast differentiation or indirectly promote it via RANKL secretion from osteoblasts [16,17,18]. Loss of NOTCH2 in osteoclast precursors impairs osteoclast formation, while ectopic NOTCH2 expression promotes it [19]. The aforementioned study revealed that in the NOTCH signaling pathway, NOTCH2 may be a key protein regulating osteoclast differentiation and bone resorption.

NOTCH2 interacts with the single transmembrane ligand DELTA/JAGGED, expressed on the surface of adjacent cells, triggering multistep hydrolysis of the NOTCH receptor by activating the ADAM10/17 complex and proteases. Thus, NOTCH2 cleaves and releases the NOTCH2 intracellular domain (ICD), which translocates into the nucleus and activates the transcription of a variety of downstream target genes, including members of the *Hes* family [20,21,22]. NUMB is a membrane adaptor protein that plays a critical role in determining cell fate and inhibiting NOTCH signaling by regulating NOTCH endocytosis/recycling and NICD ubiquitin/proteasome degradation [23,24]. NUMB is a ligand of the NOTCH receptor and can bind to components of clathrin-dependent endocytic complexes [25,26]. NUMB also contributes to the recruitment of several E3 ubiquitin ligases to the membrane-bound NOTCH2 receptor and promotes NOTCH2 degradation [27]. LNX2, a ligand of the NUMB protein, has a ring finger and PDZ domain structure containing an E3 ubiquitin ligase. LNX2 binds to NUMB and promotes its ubiquitylation, leading to the proteasome-dependent degradation of NUMB [28,29,30]. In our previous study, we reported that LNX2 expression was upregulated during osteoclast differentiation. We also found that knocking down LNX2 expression in BMMs markedly inhibited osteoclast formation, which may be caused by an increased level of NUMB, thus promoting the degradation of NOTCH2 and reducing the expression of the NOTCH2 downstream target protein HES1 [31].

Based on our previous research, we hypothesize that the LNX2/NUMB/NOTCH2 signaling axis may play a significant role in bone homeostasis. However, the role and mechanism of LNX2 in the pathological conditions of osteoporosis remain unclear. In this study, we approached the issue from the perspective of excessive bone resorption in osteoporosis. We generated a mouse model with conditional knockout (cKO) of myeloid *Lnx2* and characterized myeloid-specific *Lnx2*-deficient mice and their cellular derivatives. Our study has led to the discovery of the pivotal regulatory role of LNX2 in trabecular bone mass and estrogen deficiency-induced bone loss.

## 2. Materials and Methods

### 2.1. Generation of Myeloid Lnx2 Conditional Knockout Mice

**Figure 1:**
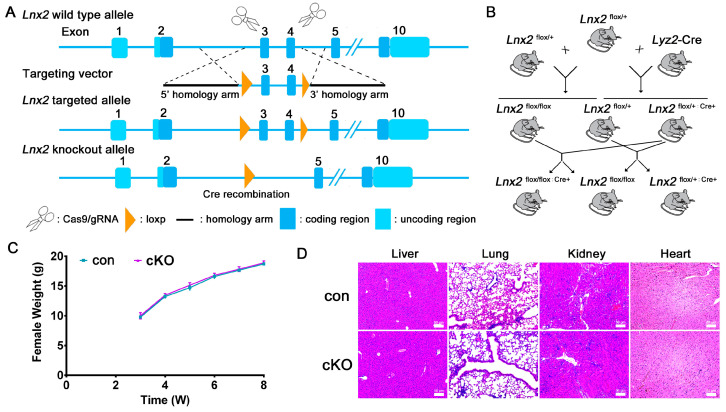
Establishment and characterization of conditional knockout mice. (**A**) Schematic diagram illustrating the construction of the *Lnx2*-flox allele. (**B**) Breeding scheme for generating *Lnx2* conditional knockout (cKO) mice by crossing *Lnx2*-floxed mice with *Lyz2-Cre* transgenic mice. (**C**) The weight curve of female mice from 3 weeks to 8 weeks old in each genotype (con, *n* = 12; cKO, *n* = 12). (**D**) H&E staining of tissue sections from control (con) and cKO mice; representative images of various tissues from each genotype are presented.

We used homologous recombination to generate *Lnx2*-floxed mice harboring a conditional allele in which exons 3–4 of the murine *Lnx2* gene were flanked by two loxP sites (Figure 1A). In brief, cas9 mRNA and gRNA were obtained by transcription in vitro; the donor vector was constructed by in-fusion cloning and contained a 4.1 kb 5′ homologous arm, a 1.2 kb Flox region, and a 4.5 kb 3′ homologous arm. F_0_ mice were obtained by microinjection of cas9 mRNA, gRNA, and the donor vector into the sperm of C57BL/6J mice. Homologous recombinant F_0_ mice were identified by long fragment PCR, and positive F_1_ mice (*Lnx2*-flox/+) were obtained by mating F_0_ mice with wild-type (WT) mice.

To obtain myeloid cKO mice, *Lnx2*-flox/+ mice were mated with *LysM*-Cre mice, and the offspring of *Lnx2*-flox/+:Cre/+ and *Lnx2*-flox/+:+/+ or *Lnx2*-flox/flox:+/+ mice were further bred to obtain *Lnx2*-flox/flox:Cre/+ (cKO) and *Lnx2*-flox/flox:+/+ (con, control) mice (Figure 1B). The genotypes of the mice were identified by short-fragment PCR.

### 2.2. Murine Osteoporosis Model Induced by Castration

A total of 24 female mice (con: 12, cKO: 12) aged 8 weeks were housed under specific pathogen-free conditions in the Experimental Animal Center of Shanghai Sixth People’s Hospital. To assess the effects of *Lnx2* on estrogen deficiency-induced bone loss, a mouse osteopenic model was established in control and *Lnx2*-null mice, as described by previous studies [32]. In brief, all mice were divided into four groups: the con/sham group, con/ovariectomized (OVX) group, cKO/sham group, and cKO/OVX group. The mice were anesthetized by 0.5% pentobarbital sodium (0.9 mL/kg weight), and then 1 cm linear bilateral lumbar lateral skin incisions were made. After exposing the muscle and peritoneum by blunt dissection, the bilateral fallopian tubes were ligated, and the bilateral ovaries were removed. The same procedure was also performed on all animals in the sham groups, but an equivalent weight of surrounding adipose tissue was removed, and the ovaries were kept intact. Then, the tissue was repositioned, and the skin was sutured carefully in layers [20]. Three months after ovariectomy, the distal femurs and L2 vertebrata were harvested for further analysis. These animal experiments were conducted in strict accordance with the guidelines of the Institutional Evaluation Committee of the Shanghai Sixth People’s Hospital Affiliated to Shanghai Jiao Tong University School of Medicine.

### 2.3. Micro-CT Scanning

Bone samples, including femurs and second lumbar vertebrae (L2), were harvested from each group of mice and fixed in 10% neutral formalin solution for 48 h. Subsequently, the specimens were scanned using a high-resolution micro-CT system (Skyscan 1176, Bruker, Belgium) with an isoresolution of 9 μm. Image analysis was performed using Scanco medical micro-CT system (Bruttisellen, Switzerland); 3D and 2D images were generated using the CTVol (v2.6.0.0) and DataViewer (v1.5.1.2) programs, respectively. The parameters of the trabecular bone, including bone volume-to-tissue volume fraction (BV/TV), trabecular number (Tb.N), trabecular thickness (Tb.Th), and trabecular separation (Tb.Sp) were computed using CTan software (v1.14.4.1) following previously described protocols [33,34].

### 2.4. Histological Analysis

Following micro-CT scanning, the femurs and L2 vertebrae were decalcified in 10% EDTA for 14 days and then embedded in paraffin. The wax blocks were cut on the sagittal plane with a slicer to produce sections of approximately 3-μm thickness. The slices were then pasted on glass slides, dewaxed with xylene, and rehydrated with a serial gradient of ethanol. Hematoxylin and eosin (H&E), TRAP, and immunofluorescence staining were performed. TRAP staining was performed using TRAP kits, as described in the literature [35]. Leica microscopes were used to analyze the sections and collect representative images. To assess the extent of bone resorption, the number of claret-red particles near the resorbed trabecular bone was counted as TRAP-positive cells. The osteoclast number (Oc.N) and the percentage of osteoclast surface per bone surface (Oc.S/BS, %) were calculated [36]. For immunostaining, the following primary antibodies were used: NUMB (2756T, CST, Danvers, MA, USA, 1:200), NOTCH2 (5732S, CST, 1:200), and CTSK (ab312354, Abcam, Cambridge, UK, 1:200). Nuclei were also counterstained with DAPI. Finally, the sections were analyzed under a laser scanning confocal microscope (Leica, Wetzlar, Germany).

### 2.5. Measurement of Serum Osteoclast Markers by ELISA

Blood samples (1 mL) were collected retro-orbitally at the time of sampling. The blood samples were centrifuged, and serum was used to measure the osteoclast markers TRAP-5b and CTX-I with a kit (Novus, Littleton, CO, USA) according to a previous study [37].

### 2.6. Isolation of Bone Marrow-Derived Monocytes and Cell Culture

MEM-α culture medium and penicillin–streptomycin solution were obtained from HyClone, while fetal bovine serum was purchased from BI. BMMs were harvested from control and *Lnx2* cKO mice. In brief, bone marrow cells were collected from the tibiae and femurs of a single 6-week-old mouse of each genotype. Red blood cells were removed by treatment with erythrocyte lysis buffer for 5 min at room temperature. The isolated bone marrow cells were then seeded onto a 10 cm Petri dish and cultured in MEM-α complete medium containing 10 ng/mL M-CSF [38]. After 24 h, the suspended cells were collected by centrifugation and then cultured in MEM-α complete medium containing 30 ng/mL M-CSF for an additional 3–4 days. After the cells reached approximately 80–90% confluence, the cells were digested with trypsin/EDTA and used for further experiments. In the functional rescue experiment on BMMs from *Lnx2* cKO mice, Numb Mouse Pre-designed siRNA (HY-RS19926, MCE, Monmouth Junction, NJ, USA) was used for cell transfection to reduce Numb expression according to the instructions of the reagent.

### 2.7. Cell Proliferation Assay

Cell Counting Kit-8 (CCK-8) solution was used to examine the effect of *Lnx2* deficiency on BMM proliferation [38]. BMMs were seeded into 96-well plates at a density of 8 × 10^3^ cells per well and cultured in MEM-α complete medium containing 30 ng/mL M-CSF for 1, 3, and 5 days, with medium replacement every 2 days. At the indicated time, the absorbance was recorded at 450 nm with a microplate analyzer. The CCK-8 assay was also used to determine the cytotoxicity of different concentrations of DAPT (a γ-secretase inhibitor) on BMMs, and DAPT (0, 1, 5, 10, and 50 μM) was added to the culture medium.

### 2.8. In Vitro Osteoclast Culture and Tartrate-Resistant Acid Phosphatase (TRAP) Staining

Osteoclasts were generated by culturing BMMs (at a density of 5 × 10^5^/well in a 24-well plate) with MEM-α complete medium containing 30 ng/mL M-CSF and 100 ng/mL RANKL for 5 days. 40 μM Jagged-1 (188,204) TFA (MCE, Monmouth Junction, NJ, USA) conditioned medium was used to upregulate the expression of the Notch2 gene and to observe its effect on the osteoclast differentiation of *Lnx2* cKO BMMs. Once large multinucleated cells appeared, the cells were fixed with 4% paraformaldehyde for 15 min, followed by TRAP staining using a tartrate-resistant acid phosphatase staining kit. Three fields in three independent wells were randomly selected from each group to analyze and calculate the average area and number of osteoclasts [38].

### 2.9. Resorption Pit Formation Assay

To assess the impact of *Lnx2* deficiency on osteoclastic bone resorption, BMMs were seeded on Corning Osteo Assay Surface 24-well plates and incubated for 5 days as previously described. Then, the 24-well plate was placed in an ultrasonic cleaner and vibrated for 2 mins. A Leica inverted microscope (Wetzlar, Germany) was used to photograph the bottoms of the wells, and three random fields of view were analyzed for each well. The bone-resorbed area percentage was then quantified using ImageJ software (v1.8.0).

### 2.10. Actin Filament (F-Actin) Staining

To investigate whether the absence of *Lnx2* affects podosome belt formation in mature osteoclasts, F-actin rings were visualized by fluorescence staining. After osteoclasts had reached maturity on day 5, the cells were fixed in 4% paraformaldehyde for 15 mins. Then, the cells were permeabilized with 0.1% Triton X-100 for 10 mins and incubated with Cy3-conjugated phalloidin (1:200) for 30 mins at room temperature. The nuclei were stained with DAPI for 5 mins. Representative images were captured using a Leica fluorescence microscope (Wetzlar, Germany) and analyzed with ImageJ software.

### 2.11. Quantitative Real-Time PCR (qRT-PCR)

The inhibitory effect of *Lnx2* on the osteoclastic differentiation of BMMs was further quantified. Total RNA was extracted from BMMs from control and *Lnx2* cKO mice with 1 mL of TRIzol reagent (Invitrogen, Carlsbad, CA, USA) after the cells had been cultured for 5 days in 6-well plates. Complementary DNA (cDNA) was synthesized from the extracted RNA with a Revert Aid First Strand cDNA Synthesis Kit (Thermo, Waltham, MA, USA). Subsequent qRT-PCR was carried out employing SYBR Green detection chemistry. *Gapdh* served as the endogenous reference for data normalization. Three independent biological replicates were conducted for all assays. Relative mRNA abundance was determined via the formula 2^−ΔΔCt^ method. Primer sequences are detailed in Table 1.

### 2.12. Western Blotting

BMMs from each experimental group were plated in 6-well plates and incubated for 5 days. After three washes with PBS, total cellular protein was harvested and quantified. Protein extracts were then resolved, transferred onto membranes, and blocked per standard protocols. Guided by protein molecular weight markers, the PVDF membranes were sectioned into individual strips for specific antibody probing. The strips were incubated overnight at 4 °C with primary antibodies targeting NUMB (2756T, CST, Danvers, MA, USA, 1:1000), NOTCH2 (5732S, CST, 1:1000), HES1 (A11718, ABclonal, 1:1000), and β-ACTIN (AC028, ABclonal, Wuhan, China, 1:10,000). Following primary incubation, membranes were rinsed with TBS-T and probed with the appropriate secondary antibodies (1:1000) for 1 h at room temperature. Immunoreactive bands were visualized using enhanced chemiluminescence substrate (Beyotime, Shanghai, China) and captured with a ChemiDoc CRS imaging platform (Bio-Rad, Hercules, CA, USA).

### 2.13. Statistical Analysis

All results are expressed as mean ± standard deviation (SD). For comparisons between groups, Student’s *t*-test or one-way ANOVA followed by Tukey’s Honestly Significant Difference (HSD) post hoc test was applied, unless otherwise specified. A *p*-value of less than 0.05 was considered statistically significant. Statistical analyses were performed using SPSS 17.0 (SPSS Inc., Chicago, IL, USA).

## 3. Results

### 3.1. Generation of Myeloid Lnx2 Conditional Knockout Mice

Based on the sequences of intron 2 and intron 4 of the murine *Lnx2* gene, we designed a guide RNA, and the transcriptional output of cas9 and gRNA is shown in Figure S1A. The results of restriction enzyme EcoRI digestion indicated that the sizes of the bands should be 9.8 K, 5.6 K, and 398 bp, but the 398 bp band was not observed, possibly because of a reduced amount of this fragment (Figure S1C). A total of 97 mice (F_0_ generation) were obtained by the microinjection of oosperm. The genotypes of F_0_ generation mice were identified by long fragment PCR, and a positive F_0_ generation mouse with the correct homologous recombination (No. 15 in Figure S1D) was obtained. Four *Lnx2*-flox/+ mice (F_1_ generation) were identified by PCR and numbered 1, 6, 7, and 9 (Figure S1E). Finally, *Lnx2*-flox/flox:*LysM*-Cre/+ mice were obtained by breeding *Lnx2*-flox/flox or *Lnx2*-flox/+ male mice with *Lnx2*-flox/+:*LysM*-Cre/+ female mice (Figure 1A,B, and Figure S2).

### 3.2. Deletion of Lnx2 in Murine Myeloid Osteoclast Precursor Cells Increases Trabecular Bone Mass and Attenuates Estrogen Deficiency-Induced Bone Loss by Inhibiting Osteoclastogenesis

**Figure 2:**
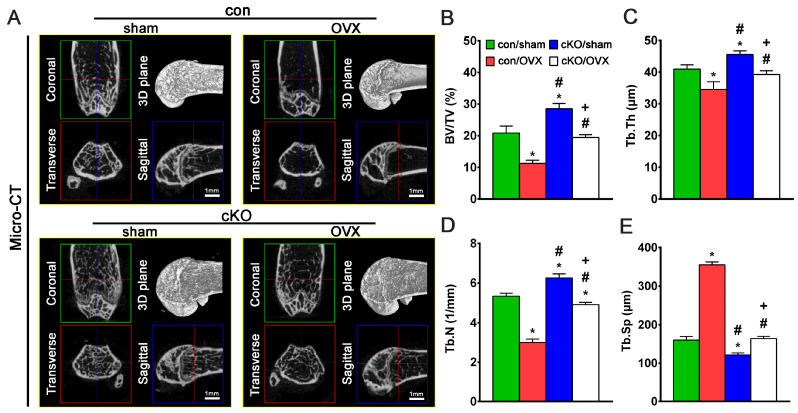
Micro-CT analysis of the distal femur in mice. (**A**) Representative CT scan images and 3D reconstructions of the distal femur from each experimental group. (**B**–**E**) Micro-CT parameters for the trabecular bone region of the distal femurs: BV/TV (ratio of trabecular bone volume to total tissue volume), Tb.Th (trabecular thickness), Tb.N (trabecular number), and Tb.Sp (trabecular separation). (*n* = 12, *, # and + represent *p* < 0.05 when compared with con/sham, con/OVX, and cKO/sham groups, respectively).

**Figure 3:**
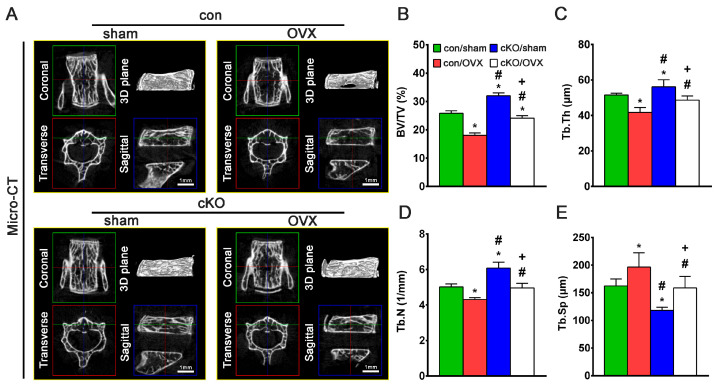
Micro-CT analysis of L2 in mice. (**A**) Representative images of the CT scan and 3D plane from each group of L2. (**B**–**E**) Micro-CT analyses of the trabecular bone compartment of L2. BV/TV, percentage of trabecular bone volume to tissue volume; Tb.Th, trabecular thickness; Tb.N, trabecular number; Tb.Sp, trabecular separation. (*n* = 6, *, # and + represent *p* < 0.05 when compared with con/sham, con/OVX and cKO/sham groups, respectively).

The *Lnx2* cKO mice were born at the expected Mendelian ratio. These mice developed and grew normally, as demonstrated by similar sizes and body weights compared to those of their littermate controls (Figure 1C). H&E staining analysis of the kidney, liver, lung, and heart indicated normal histology in these organs (Figure 1D). We next examined the bone mass and 3-D structures of 5-month-old control and *Lnx2* cKO female mice by micro-CT scanning of the distal femurs and L2 vertebrae. The coronal, transverse, and sagittal micro-CT images of both the distal femur and L2 vertebra (Figure 2A and Figure 3A, right panels) demonstrated more trabecular bone in *Lnx2* cKO mice than in their control littermates. A similar phenotype was observed in male mice (Figure S3A–E). Quantitative analysis of the micro-CT data showed a significant increase in BV/TV, Tb.Th, and Tb.N with a concomitant decrease in Tb.Sp in the femurs and L2 vertebrae of *Lnx2* cKO mice compared with control mice (Figure 2B–E and Figure 3B–E). The loss of *Lnx2* in myeloid cells had no obvious effects on cortical bone parameters in mice (Figure S3A,F,G). Estrogen deficiency induced by ovariectomy caused bone loss in both control and *Lnx2* cKO female mice (Figure 2 and Figure 3). This phenotype was less pronounced in *Lnx2* cKO mice, as a significant amount of trabecular bone was preserved in cKO mice compared to control mice.

**Figure 4:**
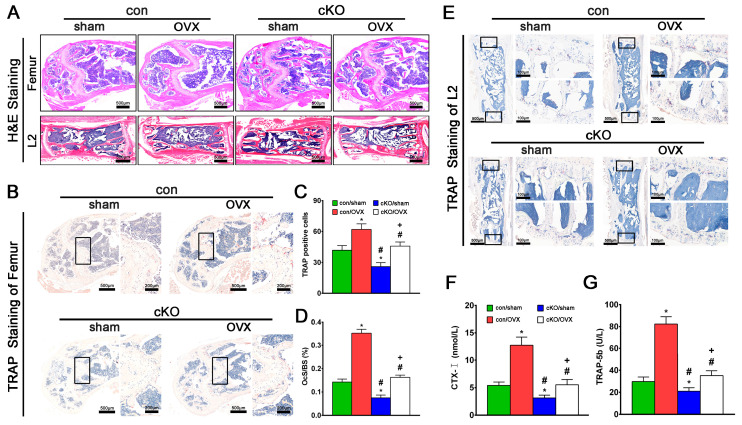
Histological staining and biochemical detection in mice. (**A**) Histological images of H&E-stained distal femur and L2. (**B**) Histological images of TRAP-stained the distal femur sections, the right side represented the enlarged image of the black box, and the osteoclasts were dyed wine red. (**C**,**D**) Quantitative analysis of the number of TRAP-positive cells and ratio of osteoclast surface to bone surface (Oc.S/BS, %) in TRAP-stained sections. (**E**) Histological images of TRAP-stained L2 sections, the right side represented the enlarged image of the black box, and the osteoclasts were dyed wine red. (**F**,**G**) The bone resorption markers CTX-I and TRAP-5b in serum were quantitatively analyzed by Elisa kits. (*n* = 6, *, #, and + represent *p* < 0.05 when compared with con/sham, con/OVX, and cKO/sham groups, respectively).

**Figure 5:**
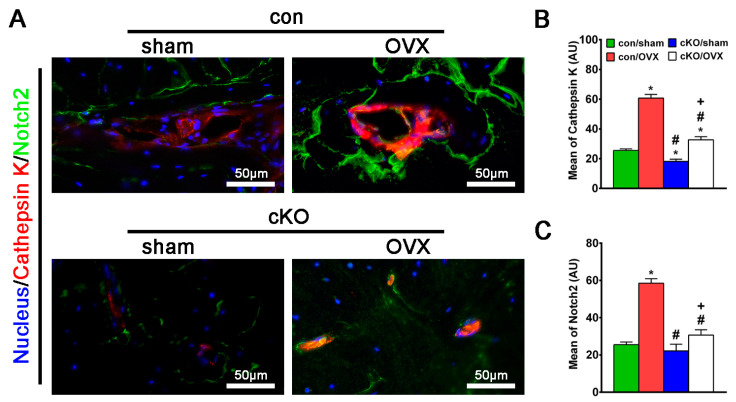
Bone tissue fluorescence double staining detection in mice. (**A**) Representative immunofluorescence images show the expression of CTSK and NOTCH2 proteins in the femoral condyle of mice. (**B**,**C**) Quantitative analysis of CTSK and NOTCH2. (*n* = 6, *, # and + represent *p* < 0.05 when compared with con/sham, con/OVX and cKO/sham groups, respectively).

Histological and histomorphometric analysis of H&E- and TRAP-stained paraffin-embedded femoral and L2 vertebral sections revealed an increase in trabecular bone in *Lnx2* cKO mice compared to control mice, similar to the micro-CT measurements (Figure 4A). The in vivo deletion of *Lnx2* in osteoclast precursor cells led to a marked reduction in both the number and surface area of TRAP-positive osteoclasts in femoral and vertebral bone tissue (Figure 4B–E). In addition, the serum expression levels of the osteoclast markers TRAP-5b and CTX-I were similar to the imaging and histological staining results (Figure 4F,G). Estrogen deficiency increased osteoclastogenesis in both control and *Lnx2* cKO mice; however, this phenotype in *Lnx2* cKO mice was less dramatic than that in control mice. We also conducted a fluorescence double staining with CTSK/NOTCH2 on the paraffin sections of distal femur tissue samples. The results in Figure 5A–C showed that in the con/OVX group, the NOTCH2 protein was highly expressed in bone collagen, and the expression of CTSK also increased, ultimately leading to the breakdown and absorption of bone collagen. In the cKO/OVX group, the expression of NOTCH2 protein and CTSK in bone collagen was significantly weakened, resulting in a reduced breakdown of bone collagen and a relative increase in bone mass. This result was consistent with the results of the CT scan and bone metabolism marker tests. Together, these findings demonstrate that *Lnx2* is essential for osteoclastogenesis and bone remodeling in vivo and that its deletion mitigates bone loss induced by estrogen deficiency.

### 3.3. Lnx2 Deficiency Has No Effect on the Proliferation of Bone Marrow Monocytes

**Figure 6:**
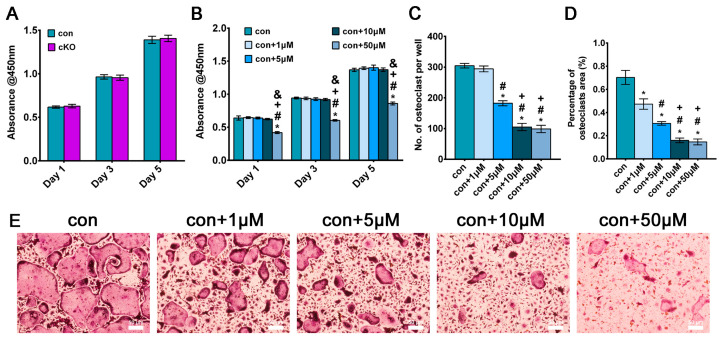
The effect of loss of *Lnx2* or DAPT on the cell proliferation of BMMs. (**A**) Cell viability was assessed using the CCK-8 assay. (**B**) The effect of different concentrations of DAPT on BMM proliferation was detected by CCK-8 assay. (**C**–**E**) Control BMMs were exposed to RANKL (100 ng/mL), M-CSF (30 ng/mL), and different DAPT concentrations for 5 days; after this period, cells were fixed and stained for TRAP. (*n* = 3, *, #, +, and & represent represent *p* < 0.05 when compared with con, con + 1 µM, con + 5 µM and con + 10 µM groups, respectively).

To evaluate the effect of *Lnx2* deficiency on the proliferation of osteoclast precursor cells, we cultured BMMs with M-CSF for up to 5 days. The number of viable BMMs was quantitatively measured by the CCK-8 assay on days 1, 3, and 5 of culture. As shown in Figure 6A, the loss of *Lnx2* had no effect on BMM proliferation at each time point. Next, we examined the dose-dependent effect of the γ-secretase inhibitor DAPT on BMM viability by the CCK-8 assay. Treatment with 1 µM to 10 µM DAPT had no effect on BMM viability; however, 50 µM DAPT significantly decreased the number of BMMs in the cultures (Figure 6B). DAPT inhibited RANKL-induced osteoclastogenesis in a dose-dependent manner (Figure 6C–E).

### 3.4. Lnx2 Deficiency Suppresses RANKL-Induced Osteoclast Formation and Osteoclast Bone Resorption In Vitro

**Figure 7:**
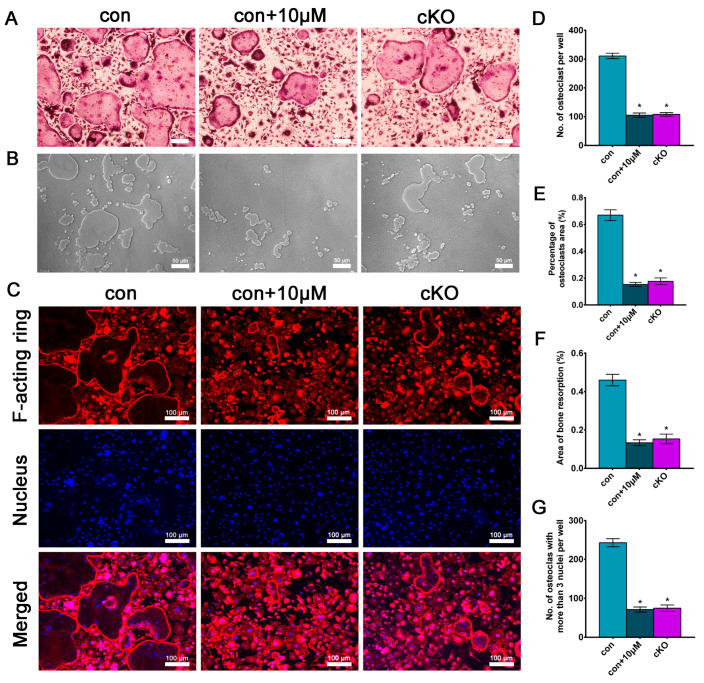
The effects of loss of *Lnx2* on osteoclast differentiation and bone-resorbing activity in vitro. (**A**) BMMs of different genotypes were stimulated with RANKL (100 ng/mL), M-CSF (30 ng/mL), and the indicated DAPT concentrations for 5 days; cells were then fixed and subjected to TRAP staining. (**B**) BMMs from different genotypes were seeded onto Corning Osteo Assay Surface 24-well plates and cultured in conditioned medium for 5 days. (**C**) The cells were stained with Cy3-labeled phalloidin and DAPI after BMMs were cultured for 5 days. F-actin rings were visualized under a fluorescence microscope. (**D**,**E**) Quantitative analysis of osteoclast numbers and areas in each group. (**F**) Eroded areas were quantified using ImageJ software. (**G**) Quantitative analysis of total osteoclasts (with more than three nuclei) per well in 24-well plates. (*n* = 3, * represents *p* < 0.05 when compared with the con group).

To determine the effects of *Lnx2* depletion on RANKL-induced osteoclastogenesis in vitro, BMMs were isolated from control and *Lnx2* cKO mice and cultured with M-CSF and RANKL for 5 days. TRAP staining showed a 3-fold decrease in osteoclast number and surface areas of *Lnx2* cKO cells and cultures treated with 10 µM DAPT compared with control cultures (Figure 7A,D,E). Using Corning Osteo assay plates to measure bone resorption, the eroded area reached 46% in the control osteoclast culture. Only 15.42% and 13.36% of the coated surfaces were resorbed by *Lnx2*-deficient and DAPT-treated osteoclasts, respectively (Figure 7B,F).

The organization of the actin cytoskeleton is crucial for osteoclast activation and function. The formation of actin-enriched podosome belts in osteoclasts cultured on plastic culture plates is a hallmark of active osteoclasts. Fluorescent Cy3-conjugated phalloidin staining demonstrated that well-organized podosome belts were formed in control osteoclasts, whereas the numbers and sizes of podosome belts in DAPT-treated and *Lnx2*-null osteoclasts were significantly reduced (Figure 7C,G). Collectively, these findings indicate that *Lnx2* contributes significantly to bone homeostasis by modulating osteoclast formation and bone resorption.

### 3.5. Lnx2 Regulates the NOTCH2 Signaling Pathway Through NUMB in Osteoclasts

**Figure 8:**
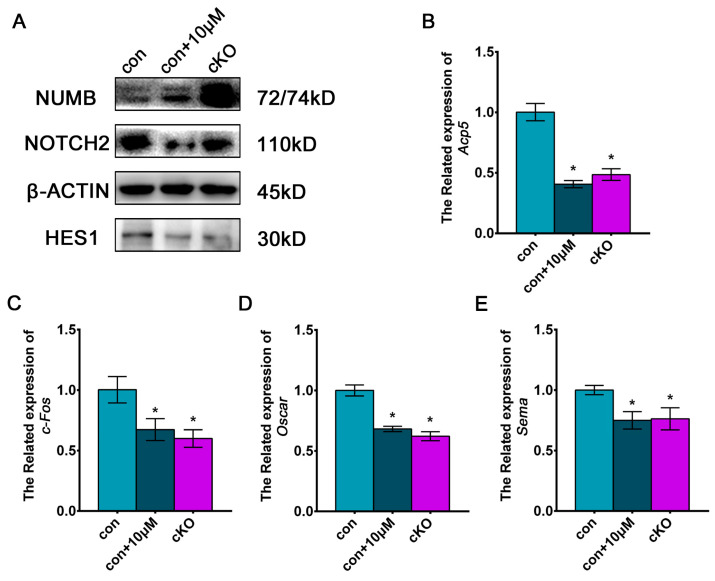
LNX2 regulates the NOTCH2 signaling pathway through NUMB in osteoclasts. (**A**) The expression of NUMB, NOTCH2 (NOTCH2 intracellular domain, NICD), and HES1 proteins was analyzed by Western blotting. (**B**–**E**) qRT-PCR results of *Acp5*, *c-Fos*, *Oscar,* and *Sema*, respectively. (*n* = 3, * represents *p* < 0.05 when compared with the con group).

To uncover the mechanisms by which LNX2 regulates osteoclastogenesis, we examined the protein expression of the LNX substrate and NOTCH antagonist NUMB in control, DAPT-treated, and *Lnx2*-knockout BMMs following 5 days of culture with M-CSF and RANKL. Previous studies have shown that LNX family proteins bind to NUMB, promoting its ubiquitination and subsequent degradation. NUMB suppresses NOTCH signaling by preventing the recycling of NOTCH receptors from endosomes to the plasma membrane in both Drosophila and mammalian systems, while also facilitating NOTCH degradation via the late endosomal/lysosomal pathway. Consequently, variations in NUMB expression affect the levels and function of NOTCH receptors. As shown in Figure 8A, NUMB protein expression was markedly elevated in *Lnx2*-deficient cells relative to control or DAPT-treated cells. As expected, the protein expression of NOTCH2 and its downstream target HES1 in DAPT-treated and *Lnx2*-deficient cells was reduced, indicating that LNX2 regulates NOTCH2 levels and activity through NUMB. In agreement with the established role of NOTCH signaling in osteoclast differentiation, as reported by our group and others [31,39], the lack of *Lnx2* and decreased NOTCH activation led to significant downregulation of the mRNA expression of osteoclast marker genes, such as *Acp5*, *c-Fos*, *Oscar*, and *Sema*, as measured by qRT-PCR (Figure 8B–E). In order to further clarify whether LNX2 regulates osteoclast differentiation and bone resorption through the NUMB/NOTCH2 signaling axes, we also conducted rescue experiments (*Numb* silencing and *Notch2* overexpression) on *Lnx2* cKO BMMs. The results of the qRT-PCR (Figure S5) showed that the low expression of *Numb* and the high expression of *Notch2* in the *Lnx2* cKO BMMs significantly enhanced the expression of osteoclast differentiation genes in BMMs, while the expression of osteoclast differentiation-related genes in the cKO group was significantly decreased. The results of TRAP and F-acting ring staining (Figure S6) also displayed that compared with the cKO group, the numbers and areas of osteoclasts in the cKO Numb siRNA group and the cKO Jagged-1 TFA group have significantly increased.

## 4. Discussion

Osteoporosis is known as the silent killer, especially in postmenopausal women, and seriously affects patients’ quality of life [40]. Mechanistic studies have revealed that a lack of estrogen in postmenopausal women and consequent abnormal osteoclast activation are common features of postmenopausal osteoporosis (PMO) [40]. Therefore, targeting osteoclast formation and bone resorption is considered a viable strategy for the treatment of osteoporosis. Despite the great efforts of scientists and doctors, few satisfactory drugs have been developed. Many potentially serious secondary effects may limit further application of the available drugs [41]. Recently, our findings, based exclusively on the use of genetically manipulated mice and their derived primary cells, indicate what we believe is the key role of LNX2 E3 ubiquitin ligase in controlling osteoclast formation and bone resorption in vitro and in vivo. Here, we showed that BMMs originating from *Lnx2* cKO mice have decreased osteoclast formation partly due to the sustained accumulation of NUMB protein, confirming that the enhancement of NOTCH2 proteolysis contributes to PMO-associated bone-protective features. Compared with the littermate OVX mice, the femoral metaphyseal and lumbar bone mass of the *Lnx2* cKO OVX mice was significantly increased. The observed severe osteoporotic phenotypes were partly due to disordered NOTCH2 accumulation in osteoclasts, as additional accumulation of NUMB reversed these phenotypes. In vitro rescue experiments, both the low expression of *Numb* and the high expression of *Notch2* were able to reverse the inhibitory effect of *Lnx2* gene deficiency on the osteoclast differentiation of BMMs. These data suggest that LNX2 plays a crucial role in bone homeostasis by regulating the NUMB/NOTCH2 signaling axis. The ubiquitin protease system may be a new medicinal target for PMO treatment.

Recent studies suggest that NOTCH signaling is a key regulator of cell fate determination during osteoblast differentiation in humans and mice [42,43], whereas the role of NOTCH in osteoclast differentiation is relatively less well understood. A recent study reported that *Notch2* mutation results in escape from FBW-mediated ubiquitination and proteolysis, resulting in osteoclast hyperactivity and Hajdu-Cheney syndrome [20]. In addition, endocytosis of the NOTCH receptor and its degradation through the ubiquitin/proteasome and lysosome also play important roles in regulating the intracellular NOTCH signaling pathway [44]. NUMB and LNX proteins are key molecules involved in NOTCH receptor degradation and signaling cascades [45]. In our previous research on LNX2, a ubiquitin E3 ligase that functions in osteoclastogenesis, we observed that LNX2 interacts with NUMB and governs NOTCH signaling in osteoclasts. Interestingly, shRNA-mediated knockdown of LNX2 expression in macrophages significantly reduced the formation of osteoclasts in vitro [31]. To better understand the effect of LNX2 on osteoclastogenesis and bone resorption in vivo and in vitro, we designed and bred cKO mice with specific *Lnx2* deletion in myeloid osteoclast precursor cells by crossing *Lnx2*-flox/flox mice with *Lyz2*-Cre mice. Here, we further elucidated the molecular mechanism by which LNX2 regulates the NOTCH2 signaling pathway and thus regulates osteoclast activity. In cKO macrophages, our data showed that *Lnx2* deficiency led to increased NUMB protein levels (Figure 8A), which may stem from reduced proteasomal degradation. In addition, a large amount of NOTCH2 protein accumulation was observed in con/OVX mice with severe osteoporosis, especially in the distal part of the femoral medullary cavity. Consistent with these findings in vivo, we also observed that *Lnx2* deletion contributed to the accumulation of NUMB, which increased the degradation of the NOTCH2 protein, resulting in decreased osteoclastogenesis and bone resorption in vitro. Therefore, these findings suggest that decreasing *Lnx2* expression or blocking LNX2 binding with NUMB is a potential strategy for the treatment of osteoporosis.

Proteolysis mediated by ADAM family enzymes and the γ-secretase complex has been proven to play an important role in NOTCH signal activation [46]. Many studies have demonstrated the efficacy of the specific NOTCH signaling inhibitor DAPT (a γ-secretase inhibitor) against massive bone resorption in mice with Hajdu-Cheney syndrome, suppressing joint destruction in osteoarthritis mice, and inhibiting inflammatory bone resorption [47]. Notably, our study demonstrated the effect of DAPT against excessive osteoclastogenesis and bone resorption in vitro. Previous studies have shown that NOTCH signaling is a potential therapeutic target for various bone resorption disorders. Except for inhibitors of the NOTCH receptor, targeted NOTCH degradation may be another strategy to inhibit NOTCH activation. NUMB is a highly conserved protein that plays a key role in determining cell fate. NUMB regulates the intracellular trafficking of the NOTCH ICD by binding to the NOTCH receptor and the clathrin-dependent endocytic complex and recruits the E3-ubiquitin ligase to the membrane-bound protein, contributing to the polyubiquitination and degradation of the NOTCH ICD [21], thereby downregulating NOTCH signaling. The LNX protein is the first member of the E3 ubiquitin ligase family with a PDZ structure and was originally identified as the interacting protein of NUMB [48]. LNX protein can bind ubiquitin and NUMB to enable the protease-dependent degradation of NUMB [49], thus enhancing the NOTCH signaling pathway. In our previous study, reducing the expression of LNX2 in monocytes contributed to the accumulation of the NOTCH inhibitor NUMB, resulting in decreased NOTCH2 levels and activity [31]. In this study, we further uncovered that *Lnx2* deficiency led to increased NUMB expression and decreased expression of NOTCH2 and its downstream protein HES1. Therefore, LNX2 can regulate the formation of osteoclasts and bone resorption by regulating NOTCH2 levels via NUMB. The ubiquitination-mediated LNX2/NOTCH2 signaling pathway may be a potential therapeutic target for osteoporosis.

Although this study reveals the regulatory role of the LNX2/NUMB/NOTCH2 axis in osteoporosis, due to resource constraints, the number of animal experiments conducted is relatively small, and no rescue experiments in vivo were performed for verification, which may have a slight impact on the experimental results. Furthermore, more in-depth research is still needed in the future to thoroughly analyze the specific mechanism by which LNX2 regulates bone resorption through the NOTCH2 signaling pathway and other NOTCH proteins.

## 5. Conclusions

In summary, we demonstrated that *Lnx2* played a critical role in osteoclast formation and bone absorption. Consistently, we found that the loss of the ubiquitin E3 ligase LNX2 in myeloid osteoclast precursor cells markedly increased trabecular bone mass and attenuated estrogen deficiency-induced bone loss. Our study suggests that *Lnx2* deficiency contributes to skeletal protection without cell proliferation disorders. The high incidence of various clinically brittle fractures and osteoporotic pain associated with estrogen deficiency is becoming one of the most common public health problems. This study provides inspiration for the development of treatment methods for patients with osteoporosis.

## Figures and Tables

**Table 1:** Primers used in the qRT-PCR of BMM cells.

Gene	Primer Sequence (F, Forward; R, Reverse; 5′−3′)
*Numb*	F: GAAGAGGCTGAGTGGCTGA
	R: GCTTCTTGATGGTGCTGGT
*Notch2*	F: CACATCAGGCCTTAATTGTG
	R: ACCTCTGGCCTGTGAATCC
*Acp5*	F: CACTCCCACCCTGAGATTTGT
	R: CCCCAGAGACATGATGAAGTCA
*c-Fos*	F: CCAGTCAAGAGCATCAGCAA
	R: AAGTAGTGCAGCCCGGAGTA
*Oscar*	F: CTGCTGGTAACGGATCAGCTCCCCAGA
	R: CCAAGGAGCCAGAACCTTCGAAACT
*Sema*	F: TAAAGTGAATGAAACCATTTGT
	R: GTCTGTGAAATGTTTTACAGTGT

## Data Availability

The original contributions presented in this study are included in the article/Supplementary Materials. Further inquiries can be directed to the corresponding authors.

## Supplementary Materials

The following supporting information can be downloaded at https://www.mdpi.com/article/10.3390/biomedicines14061180/s1. Figure S1: Generation of *Lnx2*-flox/+ mice; Figure S2: PCR results of some reproductive mice; Figure S3: Micro-CT analysis of distal femur in mice; Figure S4: Immunofluorescence staining of the distal femoral tissue sections of mice; Figure S5: The effects of silencing the *Numb* and enhancing the expression of the *Notch2* on the osteoclast differentiation of *Lnx2* cKO BMMs by qRT-PCR; Figure S6: The effects of silencing the *Numb* and enhancing the expression of the *Notch2* on the osteoclast differentiation of *Lnx2* cKO BMMs by TRAP and F-acting ring staining in vitro.
