# Supplementary material for: Myeloid-Specific Deletion of Lnx2 Attenuates Estrogen-Deficiency-Induced Bone Loss by Inhibiting Osteoclastogenesis via the NUMB/NOTCH2 Axis
Source: Biomedicines. 2026 May 22;14(6):1180. doi: 10.3390/biomedicines14061180 (PMC13295836; doi:10.3390/biomedicines14061180)
Supplement: Supplementary file 1 [file biomedicines-14-01180-s001.zip › biomedicines-4249643 - Supplementary Materials.pdf]

Supplementary Informations

Myeloid-specific deletion of *Lnx2* attenuates estrogen-deficiency induced bone loss by inhibiting osteoclastogenesis via the NUMB/NOTCH2 axis

Wei Wang[1,2], Jinhui Zhao[1,2], Ang Li[1,2], Chen Chen[1,2], Weitao Jia[1,2,], Xiaolin Li[1,2,*]*

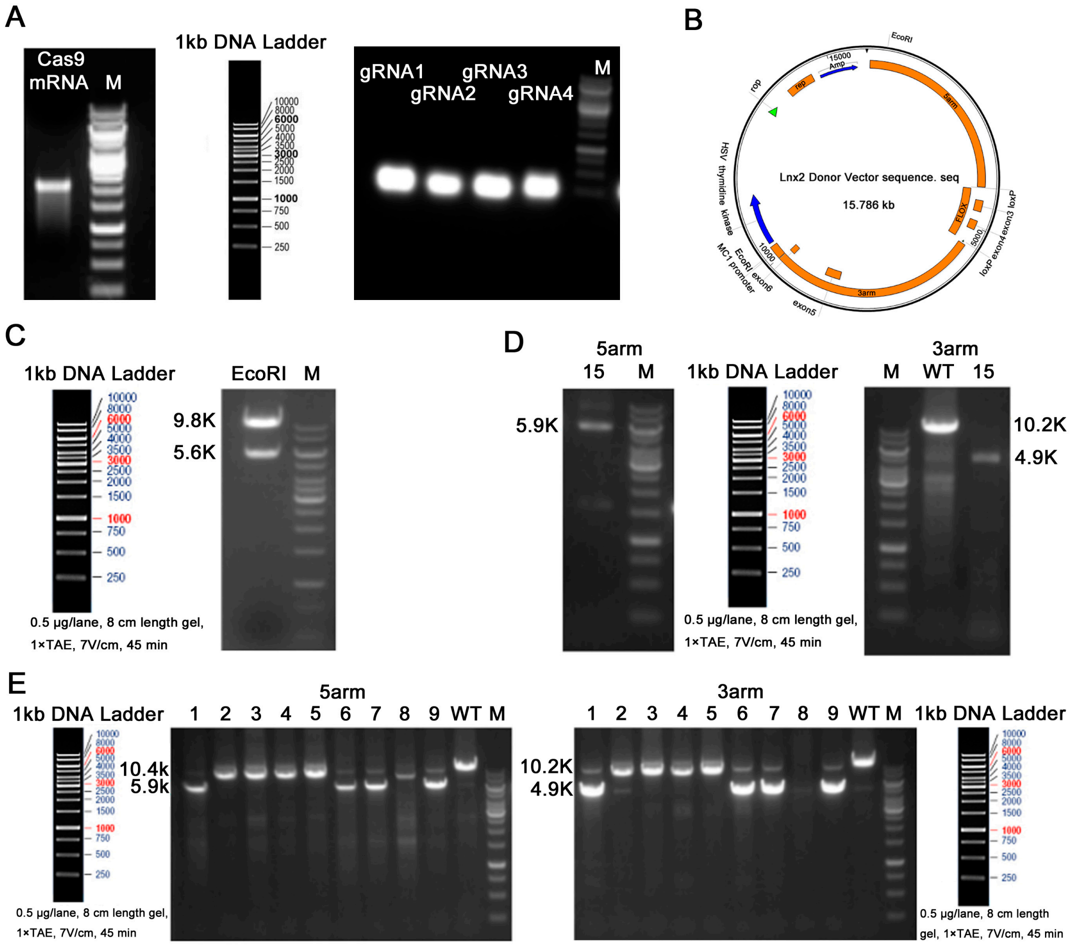

Figure S1. Generation of *Lnx2*-flox/+ mice. (A) In vitro transcriptional results of Cas9 and gRNA. (B) Homologous recombinant plasmid map. (C) Identification of recombinant plasmid enzyme cutting electrophoresis diagram. (D) Electrophoresis diagram of homologous recombination positive F_0 generation mice identified by PCR. (E) Electrophoresis maps of 5 'homologous arm and 3' homologous arm of F_1 generation mice by PCR (Number: serial number of F_1 generation mice; WT: wild-type; M: 1kb DNA ladder).

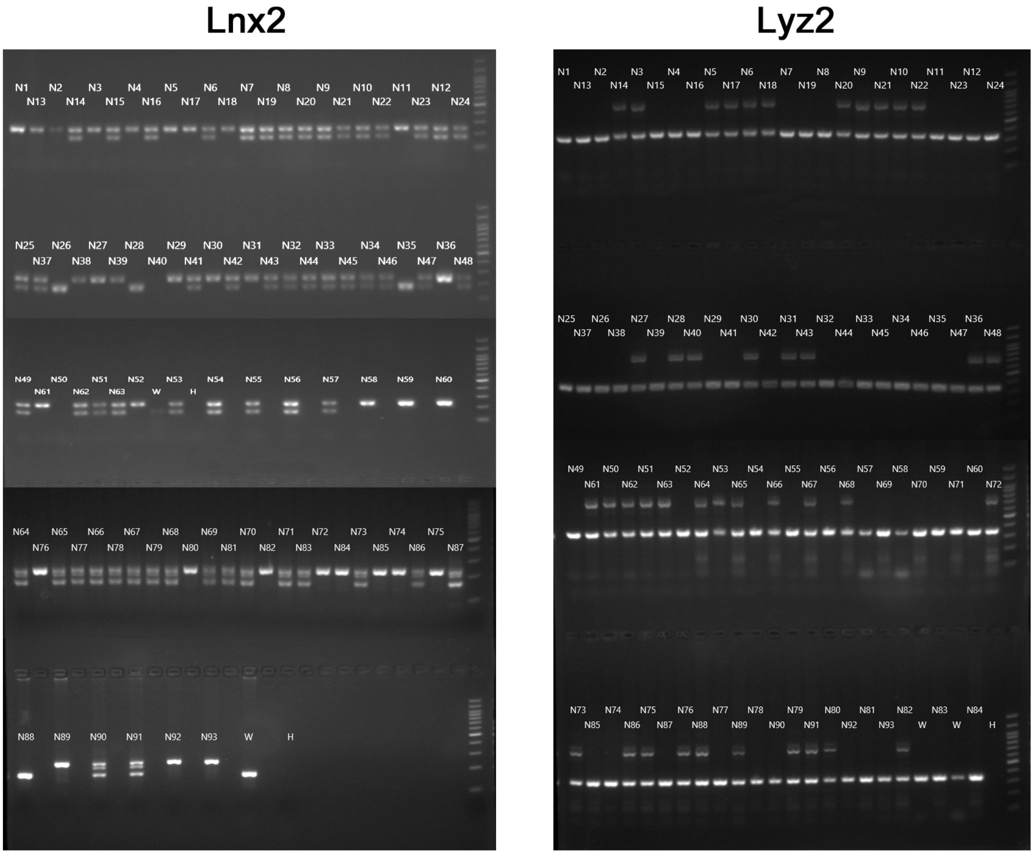

Figure S2. PCR results of some reproductive mice.

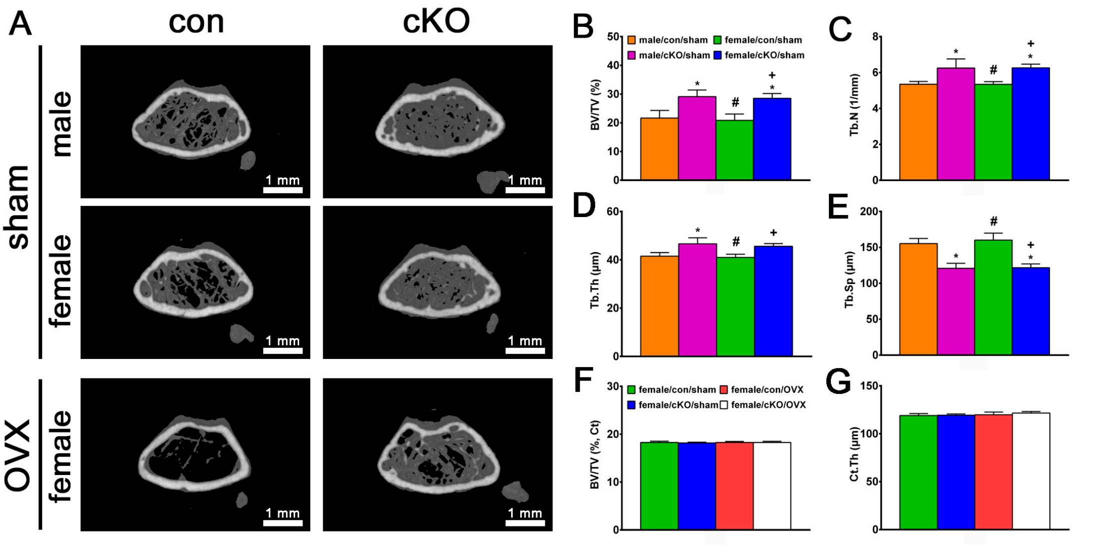

Figure S3. Micro-CT analysis of distal femur in mice. (A) Representative images of 3D plane from each group of distal femur; the white and gray represented cortical bone and cancellous bone, respectively. (B-E) Micro-CT analyses of the trabecular bone compartment of the distal femurs. BV/TV, percentage of trabecular bone volume to tissue volume; Tb.N, trabecular number; Tb.Th, trabecular thickness; Tb.Sp, trabecular separation. (F-G) Micro-CT analyses of the cortical bone compartment of distal femurs. BV/TV, percentage of cortical bone volume to tissue volume; Ct.Th,

cortical thickness. (n=6, *, # and + represent $p < 0.05$ when compared with male/con/sham, male/cKO/sham and female/cKO/sham groups, respectively.)

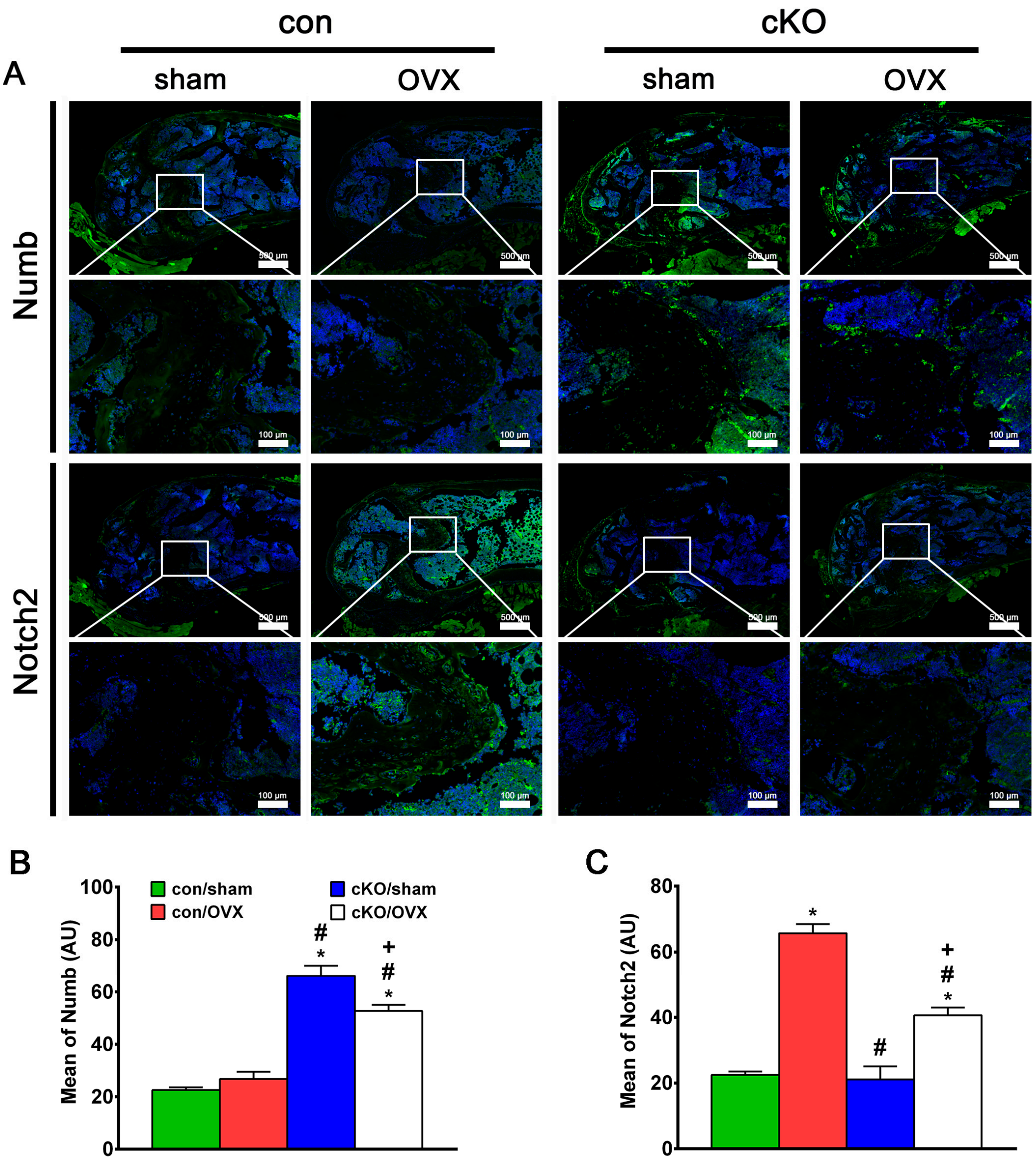

Figure S4. Immunofluorescence staining of the distal femoral tissue sections of mice. (A) Representative immunofluorescence images show the expression of NUMB and NOTCH2 proteins in femoral condyle of mice. (B-C) Quantitative analysis of NUMB and NOTCH2. (n=6, *, # and + represent $p < 0.05$ when compared with con/sham, con/OVX and cKO/sham groups, respectively.)

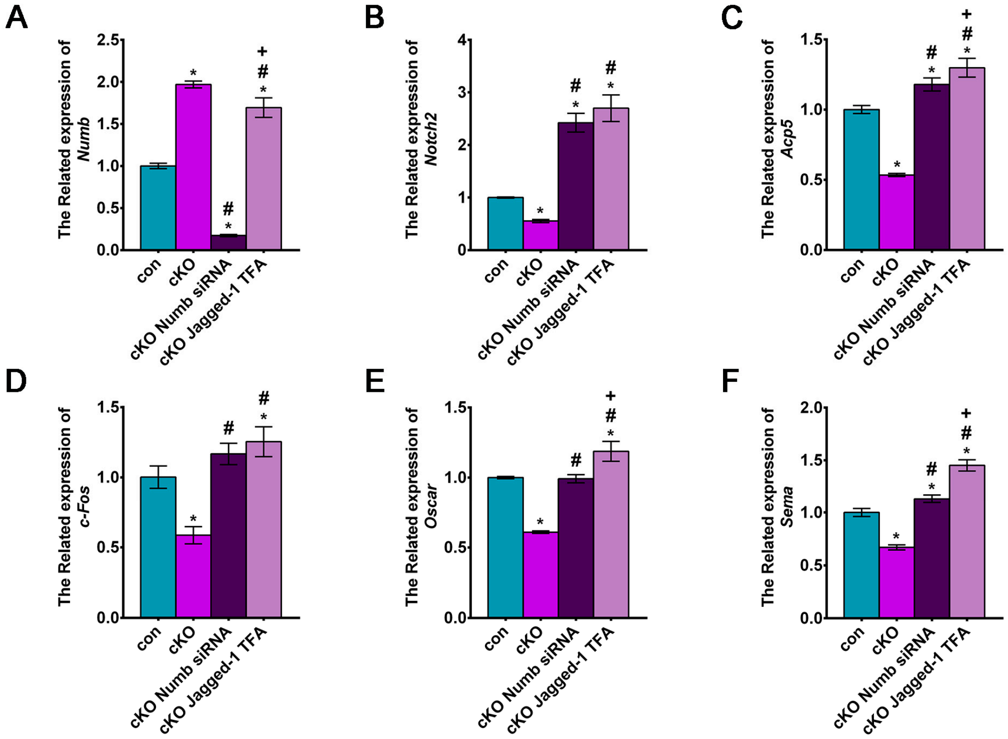

Figure S5. The effects of silencing the *Numb* and enhancing the expression of the *Notch2* on the osteoclast differentiation of *Lnx2* cKO BMMs by qRT-PCR. (A-F) qRT-PCR results of *Numb*, *Notch2*, *Acp5*, *c-Fos*, *Oscar* and *Sema*, respectively. (n=3, *, # and + represent $p < 0.05$ when compared with con, cKO and cKO Numb siRNA groups, respectively.)

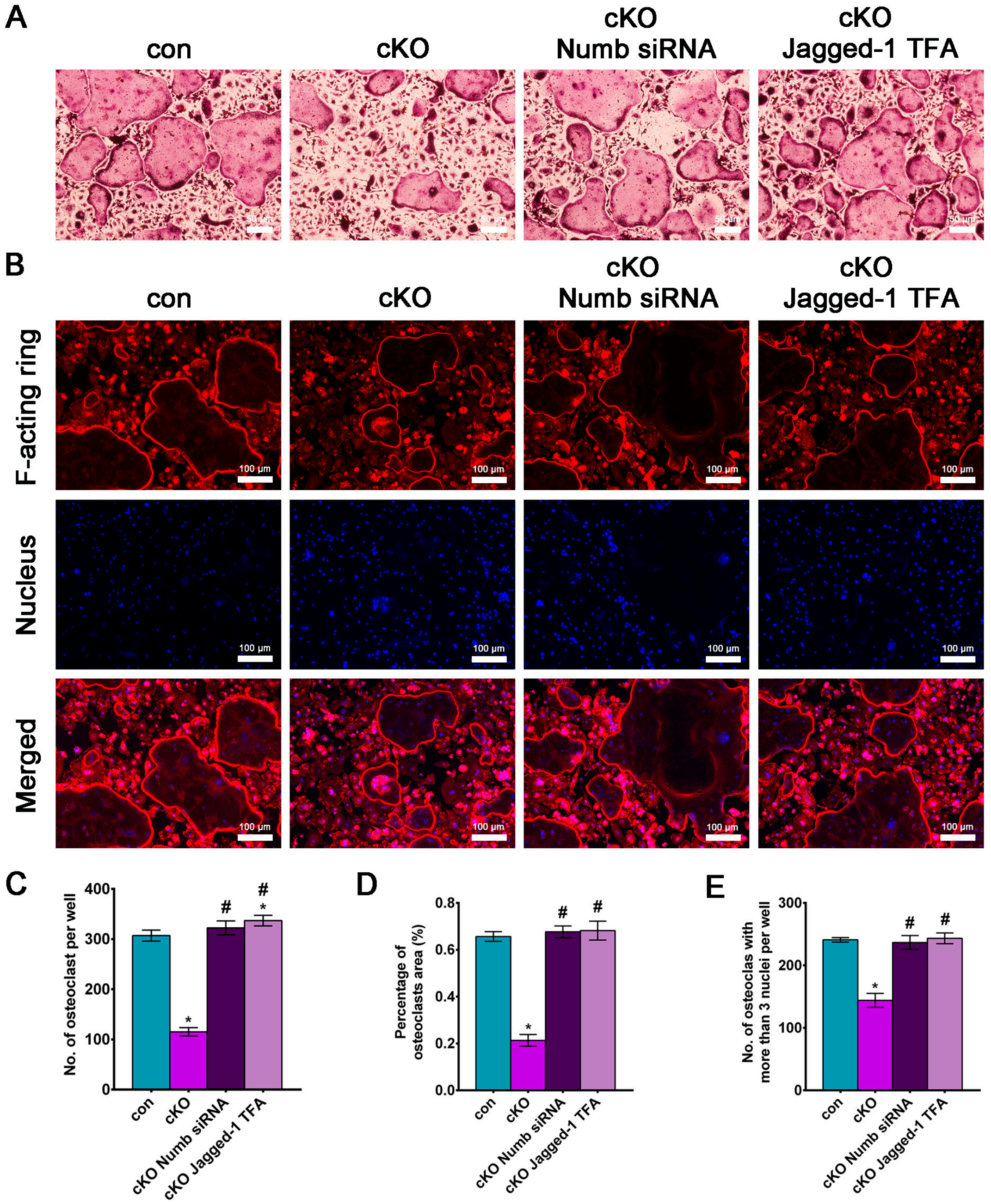

Figure S6. The effects of silencing the *Numb* and enhancing the expression of the *Notch2* on the osteoclast differentiation of *Lnx2* cKO BMMs by TRAP and F-acting ring staining *in vitro*. (A) Representative images of TRAP staining in *in vitro* rescue experiments. (B) The cells were stained with Cy3-labeled phalloidin and DAPI after BMMs were cultured for 5 days. F-actin rings were visualized under fluorescence microscope. (C-D) Quantitative analysis of osteoclast numbers and areas in each group. (E) Quantitative analysis of total osteoclasts (with more than three nuclei) per well in 24-well plates. (n=3, *, # and + represent $p < 0.05$ when compared with con, cKO and cKO Numb siRNA groups, respectively.)
